# A library for innovative category exemplars (ALICE) database: Streamlining research with printable 3D novel objects

**DOI:** 10.3758/s13428-024-02458-5

**Published:** 2024-08-02

**Authors:** Alice Xu, Ji Y. Son, Catherine M. Sandhofer

**Affiliations:** 1 Department of Psychology, University of California, Los Angeles, Los Angeles, CA 90095 USA; 2 Department of Psychology, California State University, Los Angeles, Los Angeles, CA 90032 USA

**Keywords:** Novel objects, 3D printable database, Familiarity, Similarity, Categorization

## Abstract

This paper introduces A Library for Innovative Category Exemplars (ALICE) database, a resource that enhances research efficiency in cognitive and developmental studies by providing printable 3D objects representing 30 novel categories. Our research consists of three experiments to validate the novelty and complexity of the objects in ALICE. Experiment 1 assessed the novelty of objects through adult participants’ subjective familiarity ratings and agreement on object naming and descriptions. The results confirm the general novelty of the objects. Experiment 2 employed multidimensional scaling (MDS) to analyze perceived similarities between objects, revealing a three-dimensional structure based solely on shape, indicative of their complexity. Experiment 3 used two clustering techniques to categorize objects: *k*-means clustering for creating nonoverlapping global categories, and hierarchical clustering for allowing global categories that overlap and have a hierarchical structure. Through stability tests, we verified the robustness of each clustering method and observed a moderate to good consensus between them, affirming the strength of our dual approach in effectively and accurately delineating meaningful object categories. By offering easy access to customizable novel stimuli, ALICE provides a practical solution to the challenges of creating novel physical objects for experimental purposes.

Novel objects in research contexts are items that study participants have not previously encountered. Novel objects are commonly used to study processes such as memory (Yeung et al., 2013), word learning (Kersten & Smith, 2002), and attention (Woolgar et al., 2015). These objects play a crucial role in investigating a variety of cognitive and developmental processes, as their novelty ensures that participant responses are unlikely to be influenced by pre-existing knowledge or experiences. This paper introduces A Library for Innovative Category Exemplars (ALICE) database, a resource designed to facilitate the creation of novel categories based on physical shapes through 3D printable files. Our emphasis on shape, as opposed to characteristics like color or texture, is intentional. While color can be modified during or after printing, shape is a fundamental aspect of the STL file, offering a consistent basis for research validation. This focus is underpinned by findings in category learning research, which demonstrate a tendency of children to prioritize shape when learning new words (Gershkoff-Stowe & Smith, 2004). Even if other properties, such as texture and size, are drastically different, children still readily generalize a newly learned label to objects that share the same shape (Landau et al., 1998). Moreover, most category learning studies focus on the capacity to associate a newly acquired word with objects sharing a similar shape over other properties (e.g., Vlach & Sandhofer, 2011), underscoring the importance of shape in cognitive and developmental studies. The database includes accessible and modifiable STL files for 3D printing, available at: https://4lic3x.github.io/alice_stl/.

The feature of novelty plays a pivotal role in cognitive developmental research because learning dynamics change depending on familiarity and prior experience. For example, Mather and Plunkett’s (2010) intermodal preferential looking study revealed that 10-month-old infants paid more attention to novel objects when paired with novel labels than when paired with familiar or neutral labels. In another study, Horst and colleagues (2011) used a variety of objects to examine referent mapping in 2-year-olds. They presented children with known objects, familiarized novel objects (i.e., novel objects that had been previously introduced), and “supernovel” objects (i.e., novel objects without any prior introduction). The results showed that when these young children were asked to select the referent of a novel label, they displayed a systematic bias toward choosing the supernovel objects.

The impact of novelty on attention and learning transcends early childhood, continuing to play a role in cognitive processing throughout one’s lifetime. Behavioral research highlights the beneficial effects of novelty during encoding, as evidenced by improved recognition performance (Reichardt et al., 2023). Alongside the behavioral finding, neuroscience studies have revealed that “novelty activations” (i.e., increased blood flow in the brain in response to novel images compared with familiar ones) are associated with specific brain areas, such as the right limbic regions (Tulving et al., 1996). This pattern contrasts with “familiar activations,” where the increased blood flow pattern is observed in different brain regions. Also, claiming a universal memory effect of novelty would be an oversimplification. On a perceptual level, novelty appears to be heterogeneous and exists on a continuum rather than as a binary category (Berlyne, 1960; Kafkas & Montaldi, 2018). Its impact on memory can be dose-dependent, meaning it is modulated by other factors, such as the level of unexpectedness (Frank & Kafkas, 2021; Reichardt et al., 2020).

Depending on the specific aims and design of a study, novel objects are introduced to participants either in physical form (e.g., Needham et al., 2002; Samuelson & Smith, 1998; Vlach et al., 2012) or as images (e.g., Schwab & Lew-Williams, 2016; Smith & Yu, 2008; Zettersten et al., 2018). Researchers often do not explicitly justify choosing one of these two options. Some research questions necessitate physical objects. For instance, in experiments that involve manipulating the locations (e.g., Samuelson et al., 2011) or spatial orientations (e.g., James et al., 2002) of objects, physical objects may be more relevant than images. Similarly, physical objects are also preferred in scenarios where researchers investigate tactile features of categories—such as material (Yoshida & Smith, 2005) or texture (Luna & Sandhofer, 2021; Slone & Sandhofer, 2017).

Additionally, the specific traits of study populations may limit the form novel objects take. For instance, in word-learning tasks that investigate how young children understand and apply the referents of nouns, adjectives, and sometimes verbs (Childers & Tomasello, 2002; Imai et al., 2005; Sandhofer & Smith, 2004; Taylor & Gelman, 1988), young children are often given the chance to physically interact with novel objects. This approach is taken because physical engagement, through touch and play, is more effective in maintaining young children’s interest. Sensory exploration is not only crucial for keeping children engaged, but it also aligns more naturally with their learning processes (Elkind, 2008; Ionescu & Ilie, 2018). Conversely, with infants who have limited capacity for manual exploration, researchers often employ the intermodal preferential looking paradigm (Golinkoff et al., 2013), where visual representation through pictures becomes more favorable.

Regardless of the form in which novel stimuli are presented, it can be imperative to effectively operationalize novelty to uphold the credibility of research findings. For example, research by Barrett et al. (1993), Farrar et al. (1992), and Carmichael and Hayes (2001) highlights the significant role prior knowledge plays in shaping children’s understanding of new concepts and categories. Thus, failing to validate the novelty of these stimuli properly could significantly compromise the integrity of the experiment, as prior knowledge may interfere with the learning process. Samuelson and Smith (2005) found that young children tend to naturally associate novel objects with familiar nouns, especially if these objects bear perceptual resemblances to familiar items. In fact, children with larger vocabularies learn novel labels for known referents more efficiently than their peers with smaller vocabularies (Sénéchal et al., 1995). In this case, the familiar referent may activate rich, connected word knowledge and provide additional cues during the learning of a novel label. Given these findings, the effect of prior knowledge on novel word learning cannot entirely be eliminated. However, it can be reduced by selecting objects that do not resemble familiar items to learners. These findings emphasize the importance of validating the novelty of stimuli. Such validation ensures, as much as possible, that these stimuli represent genuinely new learning experiences for participants, largely free from the influence of pre-existing associations.

Researchers have access to comprehensive databases such as the Novel Object and Unusual Name (NOUN) database, which offer validated image (and potentially physical) stimuli for research (Horst & Hout, 2016). While these databases provide a valuable repository, the process of acquiring physical counterparts to these images can be fraught with challenges. The creation or selection of novel physical objects for study purposes is a process marked by high variability and a lack of standardization. Researchers typically design and create these objects independently or choose everyday items they assume most young children have not encountered (e.g., Son et al., 2008) without standardized guidelines or shared resources. The result of this individualized approach is a diverse array of stimuli, each unique to its study, which can be seen in the varied examples across research like Horst and Samuelson (2008), Vlach et al. (2008), and Graham et al. (2010). Such variability can challenge the comparability of results (Judd et al., 2012). Additionally, the novelty of these stimuli is not always assured, as they frequently lack separate, rigorous validation experiments.

## Introducing ALICE: A 3D printable novel object repository

Recognizing the limitations inherent in the current idiosyncratic approach, and acknowledging the usefulness of existing databases (Horst & Hout, 2016), we propose a new database for printable novel physical objects—the ALICE database.

The ALICE database is an open-access resource featuring 30 novel categories, each designed for easy production with a 3D printer. The decision to start with 30 distinct shape-based categories ensures an ample variety for typical novel object learning studies, which include multiple trials often needing a target and several distractors per trial. For example, if there are six trials, and each trial has one target object in the learning phase and an additional three distractors in the testing phase of a four-choice test, 30 objects are sufficient. We created the novel objects using Shapr3D, a well-known 3D modeling software that offers a complimentary educational license. Each novel object is a fusion of three or more geometric shapes to introduce complexity. This initiative arose from the understanding that crafting unique shapes poses a significant challenge, while customizing colors and textures is a simpler task. Therefore, our goal is to provide a collection of complex, novel 3D shapes that researchers can easily adapt by altering size, color, and texture according to their needs using the printable files provided. By introducing 3D printable objects into research, we aim to facilitate the physical reproduction of systematically adaptable novel objects. To increase their practicality, we include validation results and provide files that can be edited or printed. Our goal is to contribute a comprehensive and adaptable tool to the scientific community’s research arsenal. The ALICE database is designed to encourage contribution and collaboration, allowing researchers to share their own 3D printing design files. ALICE is expected to grow as contributions accumulate, encompassing an ever-broadening variety of novel object models.

The ALICE database leverages technology to bring about multiple advantages:

Firstly, the accessibility of 3D printing services is rapidly increasing within research institutions and is expanding into public libraries, K-12 schools, and universities. This expansion can be attributed to the growing acknowledgment of their substantial contribution to educational environments (Ford & Minshall, 2019). For researchers, the advantage lies in the convenience of being able to print objects from the ALICE database as needed, in any desired quantity, and at a low cost. This is particularly advantageous for replication studies or developmental research. In the latter, there is frequently a need for multiple exemplars within the same category, typically defined by a common shape but varying on other dimensions such as size and color (for an example, see Price & Sandhofer, 2021).

Secondly, 3D-printed objects demonstrate remarkable versatility in characteristics such as size, texture, color, and material. The technology behind 3D printing involves constructing objects by sequentially layering thin slices based on a computer-generated model, with the material type varying according to the printer used (Horvath & Cameron, 2015). Advanced slicing software, like UltiMaker Cura 5.3, allows for detailed customization, enabling the production of objects with varied dimensions and textures, from smooth to rough surfaces. Typically, this level of customization is readily available through the printing service. In the ALICE database, we offer STL files that enable researchers to not only print objects but also customize them according to their specific requirements, for example, attaching accessories or altering dimensions. Furthermore, the range of 3D printing materials includes rigid options like polylactic acid (PLA) and more flexible ones like thermoplastic polyurethane (TPU). Additionally, there is a wide selection of filament colors, and researchers can also choose to color the objects post-printing. This adaptability of 3D printing can satisfy a wide range of research needs.

Thirdly, akin to established visual object stimulus databases (e.g., Brodeur et al., 2010; Horst & Hout, 2016; Migo et al., 2013), the ALICE database offers objects with standardized information on novelty and similarity. This aspect significantly reduces the time researchers might otherwise spend on preliminary studies to determine the novelty and similarity of self-created objects. Researchers using the ALICE database can efficiently choose objects tailored to their research design, selecting based on preferred object similarity. Validating the novelty of these objects will be the focus of the studies in the latter part of this manuscript.

Finally, while not our primary focus, the ALICE database also caters to researchers who need images and rotating animations, not just physical objects. Unlike many existing picture databases that present object images in a few color combinations and from restricted perspectives, which can limit their relevance to real-life learning experiences where people often see objects from multiple viewpoints, with ALICE, the included STL and OBJ files can be easily imported into 3D computer-aided design (CAD) software, such as Shapr3D, allowing for the creation of new images from any angle or the modification of colors to suit diverse research requirements.

By providing easy access to a large collection of highly customizable novel stimuli, the ALICE database enhances the efficiency of the research preparation stage that necessitates physical objects.

## The present research

In Experiment 1, adult participants were shown static images and animations depicting each of the 30 objects. Their task was to judge the novelty of each object’s shape. If the object was recognizable to them, they identified it; if the object was unfamiliar, they were asked to briefly describe its appearance. In Experiment 2, we used multidimensional scaling (MDS) to analyze the perceived similarities between objects as judged by participants through a drag-and-drop interface. This approach was in response to the stance of researchers like Horst and Hout (2016), who argue that “impossible” geometric shapes are essentially simple. Our objective was to validate the inherent complexity of our objects, with a particular focus on their shape. We anticipated that the MDS analysis would reveal participants applying a range of criteria, not limited to just one or two, in evaluating the similarity of objects based on their shape. In Experiment 3, we used two clustering methods—*k*-means clustering and hierarchical clustering—to identify objects that are sufficiently similar to each other to form distinct “global” categories. This identification is based on similarity measures derived from MDS analysis. The experiment aims to assist category learning researchers by providing objects with notable similarities.

## Experiment 1

The aim of Experiment 1 was to assess the novelty of the objects in the ALICE database. Novelty can be measured in two major ways: as a binary concept (novel or not) or as a continuum of familiarity (Horst, 2013; Kafkas & Montaldi, 2018). These two different measures of novelty might also have an important relationship to one another: as an entity becomes increasingly familiar, it categorically transitions from being merely recognizable to becoming “known” (e.g., these are entities that are not only recognized but also described with a single word; Horst, 2013). We wanted to take both these measures of novelty into account. Consequently, we asked two types of questions to capture individuals’ judgments of novelty: (1) categorical judgments of novelty, with participants simply indicating whether the object is familiar, and (2) a more descriptive approach to evaluating novelty, where participants would provide either a name or a description of the object. We posited that if participants could succinctly name or describe the object with a single concept, it would suggest familiarity rather than novelty.

### Method

#### Participants

We recruited 96 undergraduate participants from the Psychology Department Subject Pool at the University of California, Los Angeles, with our sample size slightly exceeding but largely comparable to those used in previous studies for establishing standardized picture stimuli databases (e.g., Brodeur et al., 2010, 2014; Tottenham et al., 2009). The sample comprised 81 individuals who self-identified as female, 12 as male, and three as other genders or preferred not to answer. The age range of the participants was 18 to 47 years (*M* = 20.75, *SD* = 3.79). Our sample was ethnically diverse, with 18.75% self-identifying as White, 44.79% as Asian or Asian American, 12.50% as Hispanic, Latino, or of Spanish origin, 9.38% as Middle Eastern or North African, 2.08% as Black or African American, and 6.25% as multiethnic, with the remaining participants preferring not to answer. Additionally, 71.88% of the participants were native English speakers. We excluded data from four additional participants because they did not fully engage with the experiment. For example, they provided the same descriptions for multiple distinct objects (*n* = 1), or they did not finish the experiment (*n* = 3). Informed consent was obtained from all participants in accordance with the guidelines of the university’s Institutional Review Board (IRB #23–000203).

#### Materials

**Fig. 1:**
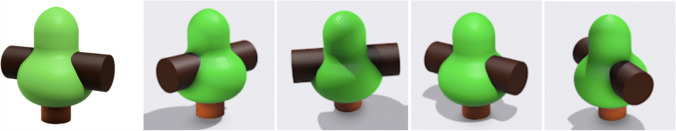
Example object image (left) and screenshots from the rotation video (right). *Note*. The original images have a transparent background. They appear to have a white background to participants because the webpage background color is white

We created a 400 × 400-pixel color image for each object in the ALICE database. We applied three distinct colors for each object, ensuring the visibility of the different components within each configuration. The images were captured from a three-quarter perspective against a transparent background, a viewpoint selected based on findings by Nonose et al. (2016) that this angle enhances object recognition and improves the quality of the view rating. Additionally, we created a 10-second video clip for each object, showing a 360-degree rotation around the transverse plane against a light grey background. The images and videos were carefully cropped to center the object and ensure it occupied approximately three-quarters of the scene. See Fig. 1 for an example object image and screenshots from its rotation video.

#### Design and procedure

Individuals from the Psychology Department Subject Pool at the University of California, Los Angeles participated in the experiment remotely, using their personal computers and a web browser. After accessing the study link, they read an introduction to the purpose of the study, were briefed on the novelty-checking tasks ahead, and consented to participate. There were 30 novelty-checking trials, one per 3D object. The order of the objects was randomized across participants.

On each trial, participants were presented with an image and a video clip of one of the 30 3D objects and asked to respond to the two questions below. Participants had the flexibility to spend as much time as needed on each object, with the requirement to answer both questions before proceeding to the next object. Participants were not able to go back to a trial once they had moved on to the next.

Question 1 was a yes/no query: “*Have you seen this category of object in your life before? Please decide based solely on the SHAPE of the object, IGNORING COLORS.*” This question aimed to capture categorical judgments of familiarity/novelty. If more participants answered “no” to this question, it would suggest greater unfamiliarity or novelty. We will refer to the percentage of “no” responses as the novelty rate.

Question 2 was designed to capture the object’s identity or, at least, the participant’s perception of it: “*If yes, what is it? If no, please provide a description of this object in a few short sentences. Please name or describe based solely on the SHAPE of the object, IGNORING COLORS.*” Presumably, if many participants identified the object with the same name or description, this suggests greater familiarity. Disagreement or variability in naming suggests novelty. The responses to this identity question were coded independently of participants’ answers to the categorical question that preceded it. For example, a participant who said “no” to the first question (they had never seen the object before) could still use a familiar label to describe the novel object (e.g., “It looks sort of like a [label]”), which may be a label shared across many participants. This discrepancy might suggest that even among participants who initially claimed unfamiliarity with the object, the use of commonly recognized labels in their descriptions indicates an underlying recognition based on the object’s shape, reflecting a shared, albeit subconscious, familiarity.

**Fig. 2:**
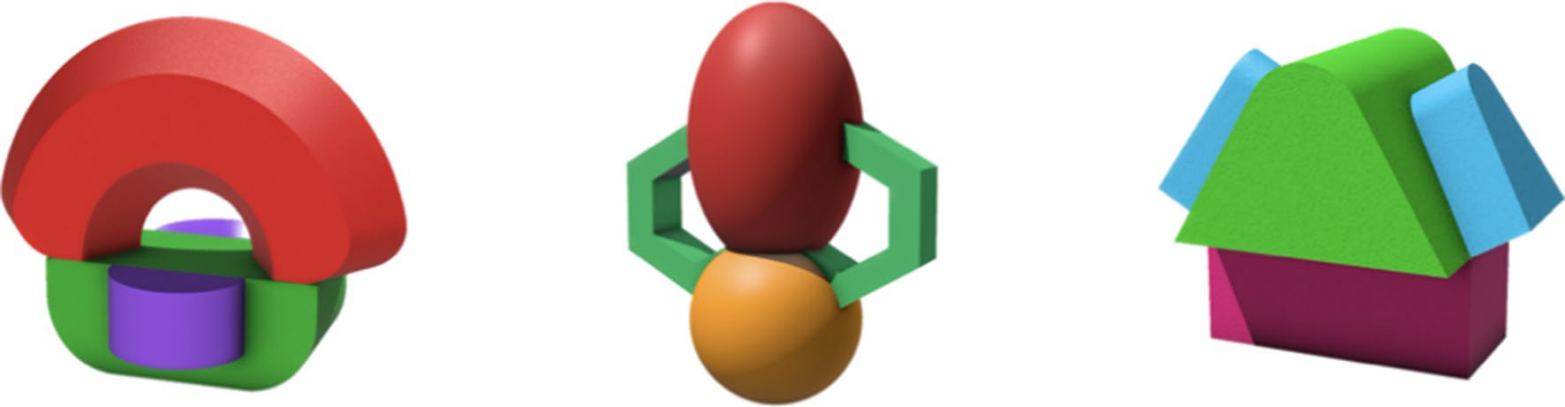
Image of Object 1 (left), Object 12 (middle), and Object 9 (right)

Consequently, we adopted a descriptive approach to evaluate novelty by considering participants’ agreement on the object’s identity through their naming or description, regardless of the participant’s response to Question 1. Specifically, we assessed this agreement for each object by comparing the most frequently mentioned “single-concept answer” to all single-concept responses. For example, if a participant described Object 1 (see Fig. 2, left) as “*looks like a rainbow*,” we coded their answer as “rainbow,” the single concept described in their response. However, another participant described Object 1 as a “*Rainbow curve over a half prism with two half circular prisms attached to either side*.” This description contained two recognized concepts: “rainbow” and “prism.” Therefore, their answer described a hybrid object (rainbow + prism) and did not qualify as a single-concept response.

We also collapsed synonyms for each concept mentioned in participants’ responses to Question 2. For example, “semicircle” and “half circle” were both coded as “semicircle.” Any spelling errors were corrected during the coding process.

To calculate the level of agreement for each object, we conservatively divided the number of participants who chose the most popular concept (e.g., “rainbow”) by the total number of single-concept responses.

### Results and discussion

The participants’ responses to Question 1 (“*Have you seen this category of object in your life before?*”) revealed varying levels of unfamiliarity with the 30 objects, with novelty rates (percentage of “no” responses) ranging from 18.75% to 68.75%. On average, the novelty rate was 44.34% (*SD* = 12.77%). For some objects with low novelty rates, participants attempted to make idiosyncratic connections between the displayed object and a familiar category. For example, one participant who said they had seen Object 12 (see Fig. 2, middle) before described it as “*a claw machine retrieving another object.*”

Indeed, when we examined the “category of object” described by the participants, the descriptions were highly variable and did not arrive at a consensus answer (more than 50% agreement) for 39 of the 40 objects*.* Among a total of 2711 names/descriptions instances provided across 30 object categories, 56.40% of them indicated a single shape-based name. Upon further investigation of these single names, we found low levels of agreement in naming/description. For all 30 object categories shown, we looked at the percentage of responses that used the most popular single name and considered this a measure of identity agreement. That is, if all participants responded with the same name (e.g., “house”) to the shown object category, the identity agreement would be 100%. Across all objects, the average identity agreement was 15.14% (*SD* = 13.14%, range = 2.08–65.62%). The object with the highest agreement was Object 9 (see Fig. 2, right), with 65.62% of participants who labeled it using a single concept, labeling it as a “house.” This agreement rate is notably higher than two standard deviations above the mean, indicating that Object 9 is an outlier. Notably, Object 9 is the only outlier among the 30 objects based on identity agreement. The object with the second highest agreement was Object 30, with an agreement mean of 32.29%, where participants labeled it as a “clover.” The object with the lowest agreement was Object 12 (pictured in Fig. 2, middle)—only two participants provided the shared label “bug” (all other names were different from each other). In total, participants provided more than 20 unique names for this object. In sum, according to the continuum of familiarity (Horst, 2013; Kafkas & Montaldi, 2018), the identity agreement for all objects falls below the 85% benchmark established by Samuelson and Smith (1999). Only one object had a somewhat higher agreement rate (Object 9, 65.62%), though it was still within the range of being considered novel. See Appendix A for a table of the aforementioned descriptive statistics.

A Spearman’s rank correlation (two-tailed) was computed to assess the relationship between the object’s novelty rate as determined through categorical judgment (i.e., the percentage of “no” responses to the first question) and the likelihood of the object being named with a single concept. It is reasonable to assume that if an object is unfamiliar, people are less likely to have a name for it. The data supported this hypothesis, indicating a negative correlation between the two variables, *r*_s_(28) = –0.73, *p* < 0.001, 95% CI = –0.81 to –0.62. We also assessed the relationship between the object’s novelty rate through categorical judgment and the agreement on identity. A negative correlation was also found, *r*_s_(28) = –0.57, *p* = 0.001, 95% CI = –0.69 to –0.42. This indicates that the more people perceived an object as unfamiliar, the less likely they were to agree on a single concept for its naming or description. These negative correlations suggest that the novelty measures are consistent with one another and are, therefore, reliable.

## Experiment 2

Experiment 2 focused on evaluating the complexity of objects in the ALICE database. In this experiment, our operational definition of “complexity” for the objects is tied to the dimensional structure revealed by the MDS analysis. Participants were tasked with arranging objects according to their perceived similarity, and the similarity ranking of each object pair was reported. This process underpins our MDS analysis, where the pairwise Euclidean distances derived from these similarity rankings serve as the basis for uncovering the dimensional configuration of objects as perceived by participants. We posit that the complexity of an object is reflected in the number of dimensions necessary to accurately represent these perceived similarities. Each dimension identified by the MDS corresponds to a distinct criterion or feature participants implicitly used in their judgments, with more dimensions indicating a higher complexity.

### Method

#### Participants

The same participants participated in Experiment 2 immediately following the completion of Experiment 1.

#### Materials

We resized the 30 images of novel objects from Experiment 1 to 60 × 60 pixels for use in Experiment 2.

#### Design and procedure

In Experiment 2, similarity ratings were obtained for each pair of objects, and MDS analysis was performed to rank all pairs based on their similarities. MDS refers to a collection of statistical techniques aimed at visually representing the relationships among observations, emphasizing the primary dimensions along which they vary (Hout et al., 2013). This approach is particularly useful in revealing patterns or structures that may not be readily apparent and has seen extensive application in psychological research (for a review, see Jaworska & Chupetlovska-Anastasova, 2009). While there are various methods for dimensionality reduction, such as principal component analysis (PCA) and *t*-distributed stochastic neighbor embedding (*t*-SNE), MDS is particularly effective in preserving pairwise Euclidean distances or dissimilarities between data points in the reduced dimensionality space. This distinction is important to note, as different methods have different strengths. For instance, PCA is primarily focused on maximizing variance and may not always maintain the exact distances between points. On the other hand, *t*-SNE is well-suited for visualizing high-dimensional data, especially for revealing clusters and local structures. However, it may not always accurately represent the global distances as MDS does. This efficacy of MDS in maintaining distances makes it a preferred choice for applications where such preservation is crucial (see Anowar et al., 2021, for a detailed comparison of dimensionality reduction algorithms).

The rationale for prioritizing the preservation of Euclidean distances stems from the complexity of similarity perception, especially in experimental settings involving novel objects. For example, in studies with young children, methodologies often rely on forced-choice tasks (Graham et al., 2010). While effective, these tasks typically involve showing only a few objects together at a time, which may not capture the nuanced perceptions of similarity people hold towards objects that are unfamiliar to them. Similarity perception is complex because it encompasses not just binary decisions of whether objects are similar or not, but also the degree of similarity, which can be subtle. Additionally, the perception of similarity can be influenced by the context in which objects are presented and how they relate to each other (Medin et al., 1997), an aspect that forced-choice tasks might overlook. Preserving the Euclidean distances allows us to quantify the degree of similarity between objects, providing richer data than forced-choice tasks alone. Understanding these nuances is critical because young children’s ability to categorize and conceptualize novel objects can be significantly influenced by their perceptions of similarity, which in turn affects their learning and cognitive development.

To efficiently gather similarity ratings for every possible pair, we employed spatial arrangement tasks akin to those used in prior studies (Goldstone, 1994; Horst & Hout, 2016; Kriegeskorte & Mur, 2012). In these tasks, participants were presented with a subset of objects in each trial and instructed to arrange them on the screen via a “drag-and-drop” method. Following Experiment 2, we collected demographic information from the participants.

##### Spatial arrangement task

After completing Experiment 1, participants immediately proceeded to Experiment 2. They were informed that the new task involved spatially arranging multiple objects on a virtual white canvas. The instructions specified that participants could click and drag each object to move it freely. The primary objective was to rearrange these objects by positioning similar ones (based on shape, not color) closer together. Participants were informed that this task would be repeated six times, each with a different set of objects, and that some objects might recur in multiple trials.

**Fig. 3:**
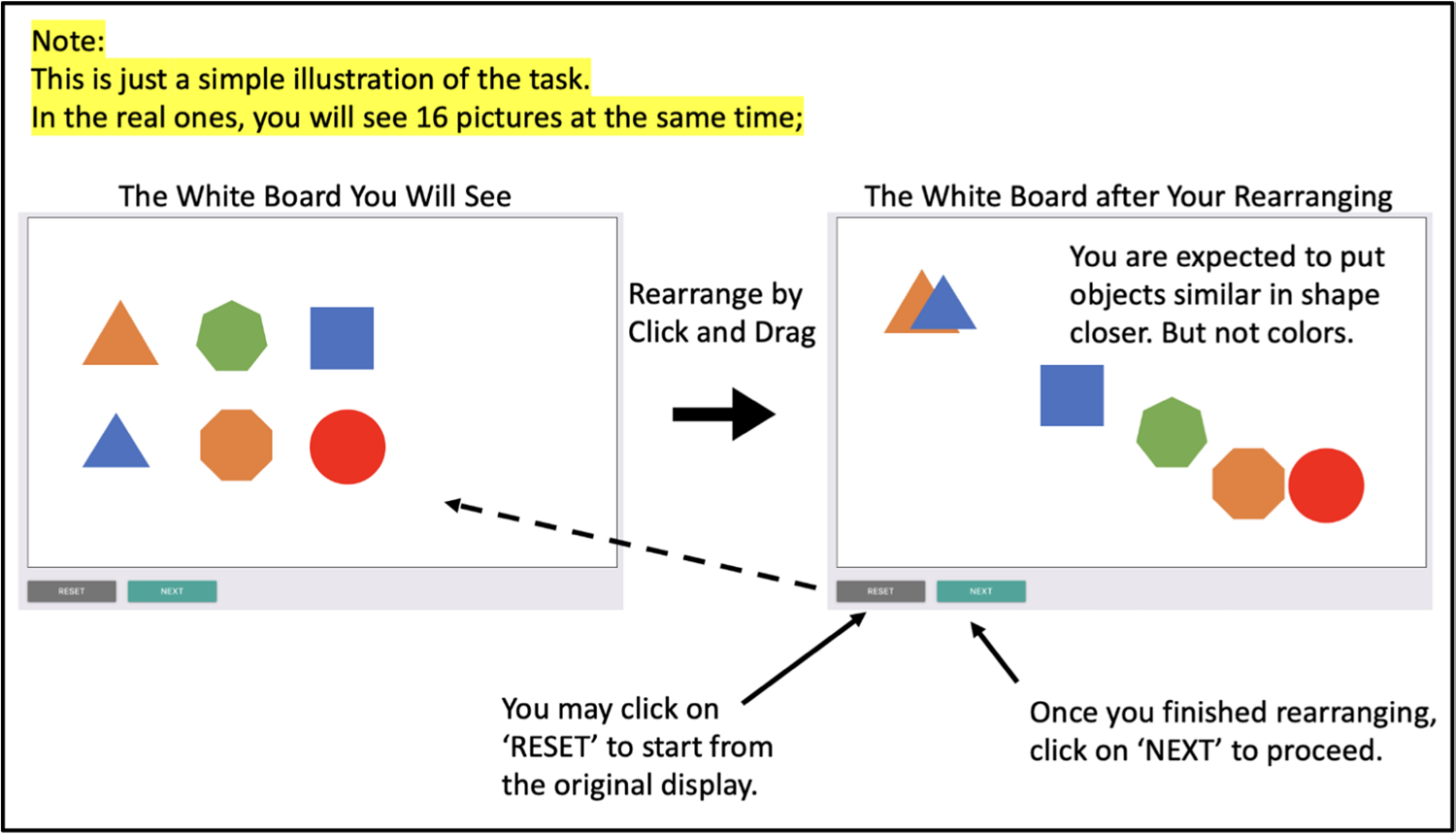
Screenshot of Experiment 2 example page

To help participants understand the task more concretely, an example page (Fig. 3) was provided after the instructions. This example consisted of two screenshots displayed side by side. The left screenshot depicted an initial arrangement of six images on a white canvas, accompanied by written instructions to rearrange the pictures such that the distance between each pair accurately represented their similarity in shape rather than color. The objective was to place objects perceived as more similar in closer proximity. The right screenshot illustrated a possible arrangement of the objects, demonstrating an appropriate way to group them based on perceived shape similarity.

**Fig. 4:**
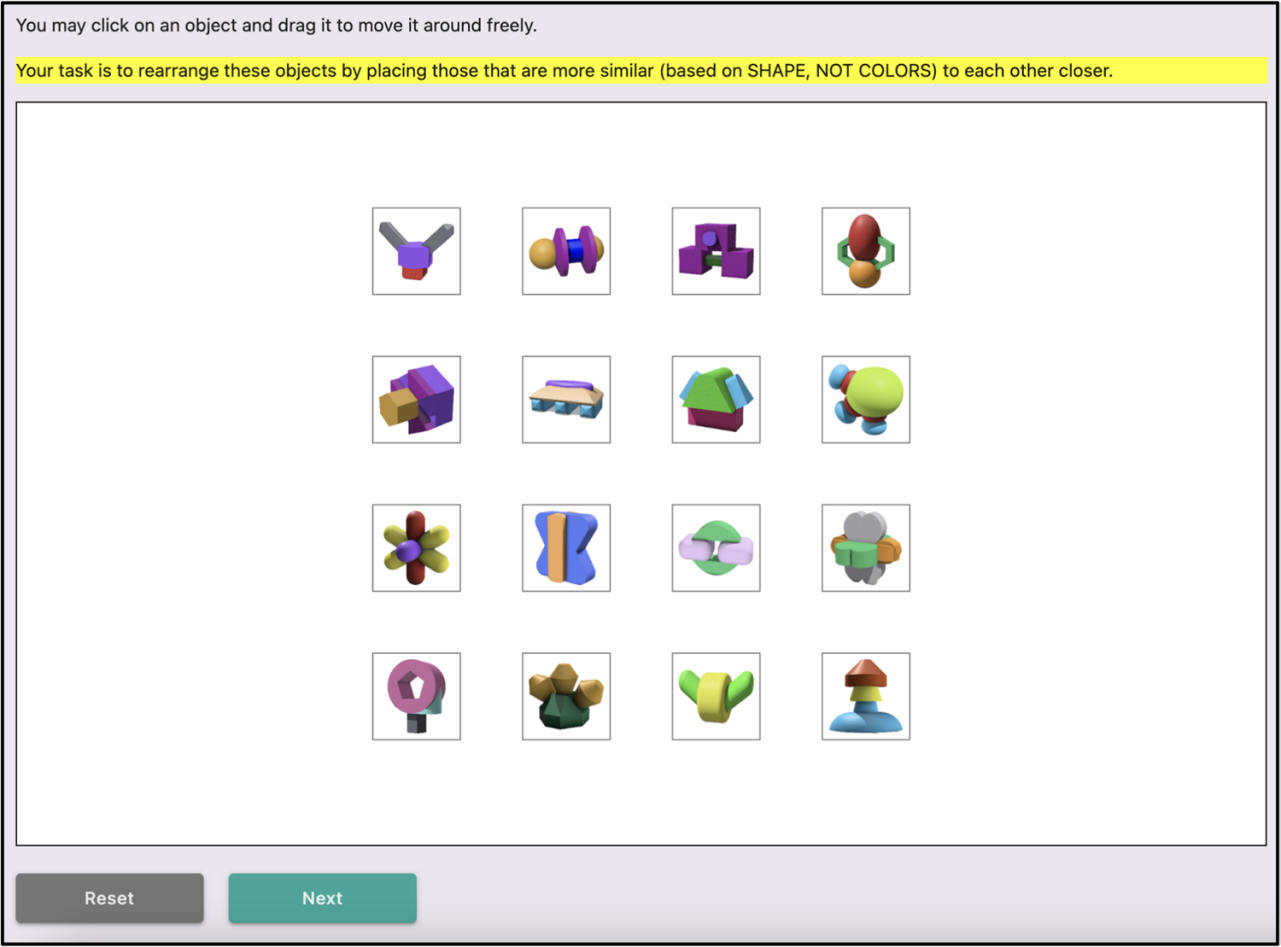
Canvas initial layout for a single trial of the spatial arrangement task

After reviewing the example, participants began the spatial arrangement task. This task involved arranging a total of 30 objects over six trials, with each trial displayed on a separate page. In every trial, participants were presented with 16 different objects organized into four rows on a 1000 × 600-pixel white canvas (see Fig. 4). Participants were given unlimited time to arrange these objects on the canvas. During this process, they were allowed to reset their arrangement to the initial state. Participants were not able to go back to a trial once they had moved on to the next.

In each trial, we recorded the final *x*- and *y*-coordinates of every object and computed the Euclidean distance (measured in pixels) between the centers of each object pair. To capture all 435 pairwise Euclidean distances (^n^C_r_, where *n* = 30 and *r* = 2), we employed a novel algorithm (detailed in Appendix B) that produced six blocks containing 16 different objects each. This algorithm used two parameters—*N* (the total number of objects, which is 30) and *K* (the number of objects per trial)—to determine the number of trials required based on the block size. To strike a balance between the number of trials and the number of objects presented in each trial, between the number of trials and objects presented in each trial, we conducted 1000 simulations for varying *K* values, from 8 to 19. The simulation results led us to choose *K* as 16, a number that necessitated a minimum of six trials to encompass all 435 unique pairwise Euclidean distances. We randomized the pairing of objects and assigned numerical identifiers (ranging from 1 to 30) for each participant. This approach guaranteed that every unique object pair was evaluated for similarity by all participants at least once. For the complete dissimilarity matrix, if a specific pair of objects had their distance measured more than once across trials by a participant, we used the average pairwise Euclidean distance for that object pair.

#### MDS analysis

We conducted an MDS analysis on the dissimilarity matrix using the MDS class from Python’s scikit-learn package (Pedregosa et al., 2011) employing the SMACOF (Scaling by MAjorizing a COmplicated Function) algorithm (de Leeuw & Mair, 2009). We opted for the metric method for two reasons. First, the distances we recorded are meaningful Euclidean distances at the interval level. This implies that the closer participants placed two objects, the more similar they are perceived to be. This is in contrast to nonmetric MDS, which is used when meaningful distances are absent and focuses on ordinal similarity (Hout et al., 2013). Second, while the nonmetric method is preferable in cases with missing entries, our study captured the Euclidean distances across all possible object pairs, leaving no missing observations in our dissimilarity matrix.

To determine the optimal dimensionality for the MDS space, we focused on assessing the goodness of fit between the distances among points in the high-dimensional space and the distances in the low-dimensional space. The stress function, designed as the discrepancy between the raw distances in the original space and the distances in the MDS space (notated as ${\Sigma }_{i<j}={d}_{ij}(X)-{\widehat{d}}_{ij}(X)$, where $X$ represents the set of coordinates of the input objects in the MDS space), serves as our primary metric for this assessment. In this context, ${d}_{ij}(X)$ is the raw average distance between object $i$ and $j$, and ${\widehat{d}}_{ij}(X)$ denotes the average distance between these objects in the MDS space. The greater the stress, the greater the dissimilarity between the MDS approximated distances and the raw distances, signaling a poorer fit. We opted not to normalize these distances because our goal was to model the distances as closely aligned as possible to the original distances.

To minimize the effect of random fluctuation (stemming from various starting configurations of the MDS space) on the selection of the optimal dimension, we simulated the stress computation 1000 times for each dimensionality under consideration. This approach allowed us to average these stress values, thereby reducing the variability attributable to different starting configurations.

Following this preparatory work, we constructed a scree plot to visualize the relationship between the number of dimensions and the average stress values from our simulations. This plot is instrumental in identifying the “elbow” point, where additional dimensions cease to significantly improve the model’s fit to the data (Jaworska & Chupetlovska-Anastasova, 2009). The selection of the optimal dimensionality is critical for balancing the fidelity of the distance approximations with the interpretability and simplicity of the MDS representation.

Having determined the optimal dimensionality, we employed the multiple-random-starts approach, a common heuristic strategy supported by scikit-learn, to choose the simulation that yields the lowest stress, thus best representing the original distances. Alternatively, there are other options for determining the starting configuration, such as the Simplex and Torgerson methods. For a comparison of these methods, refer to Horst and Hout (2016).

### Results and discussion

#### Distance/dissimilarity matrix

**Fig. 5:**
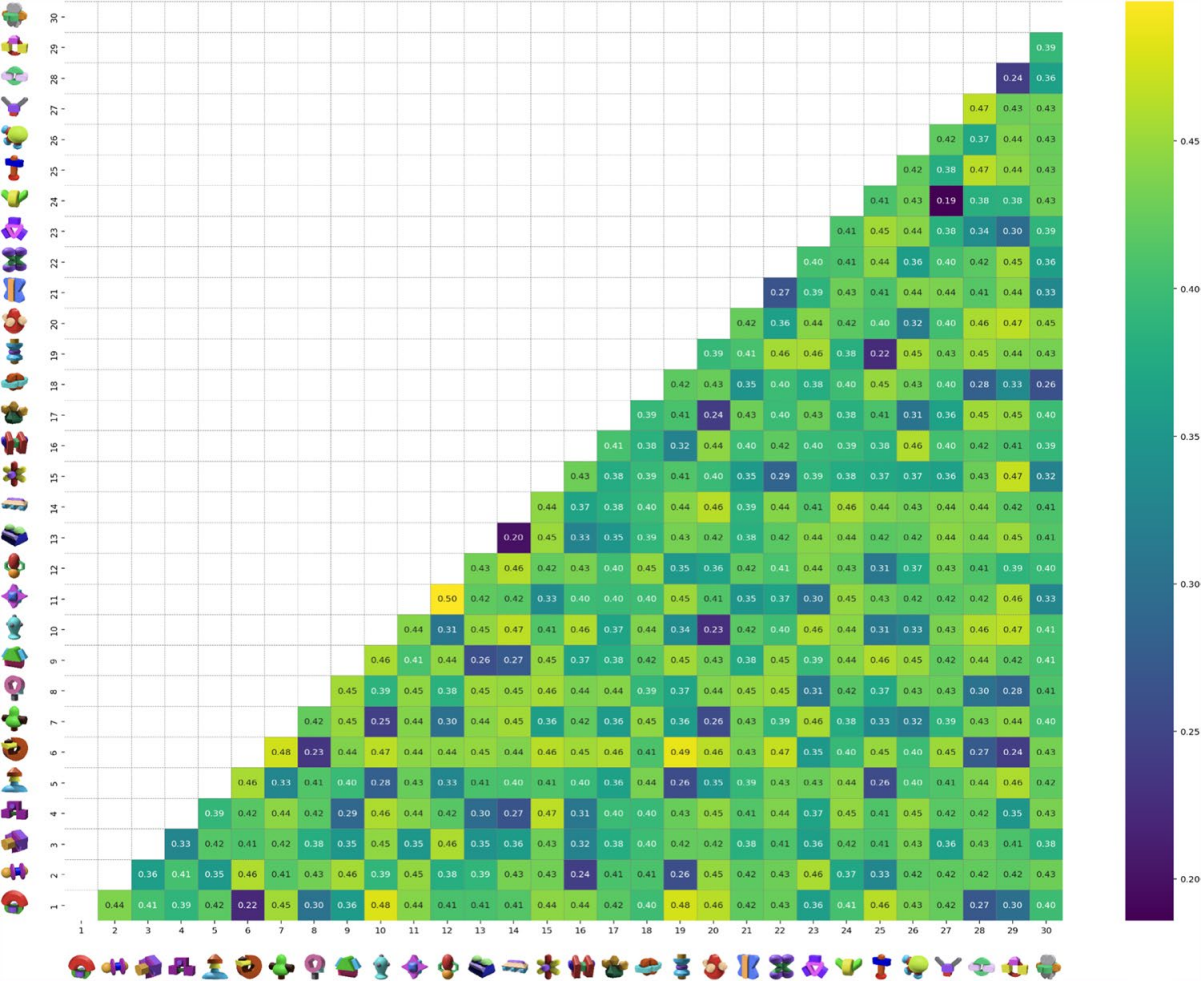
Distance/dissimilarity matrix. *Note*. This figure presents the distance/dissimilarity matrix, constructed using normalized coordinates. Each cell in the matrix represents the dissimilarity value between a pair of items, with smaller values (indicated by darker shades) signifying greater similarity (i.e., shorter raw distance). The scale of dissimilarity is symmetric—hence, only the lower triangle of the matrix is displayed to avoid redundancy

The distance matrix, constructed using the normalized coordinates, is shown in Fig. 5. A larger value (indicated by a lighter color) suggests that the pair of items is less similar to each other. We excluded the upper triangle of the distance matrix because the similarity between an object pair is treated as symmetric in our context.

#### Correlation between color and similarity judgments

**Fig. 6:**
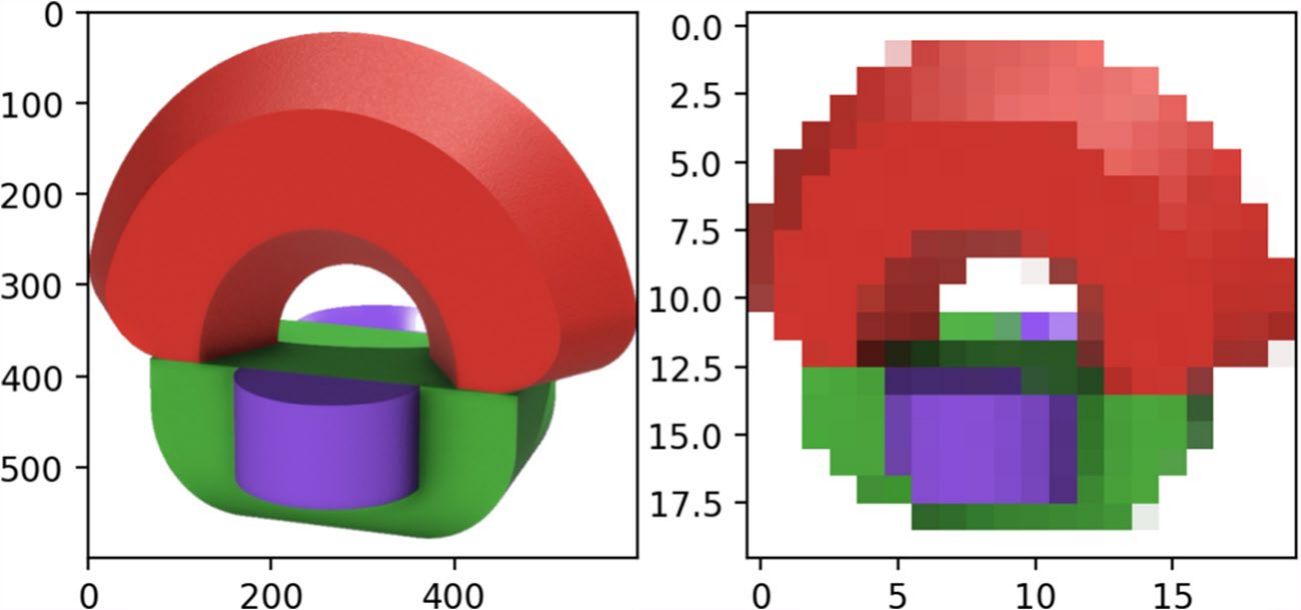
Example of original (left) and low-resolution (right) object image. *Note*. The image on the right is an illustration of the 20-by-20-pixel image. For visibility, it has been enlarged while maintaining the same proportions

Because our stimuli featured a diverse palette of colors, we tested whether participants’ similarity judgments were influenced by color, as our primary focus was on shape similarity. To explore this possibility, we needed a measure of color similarity across our objects. We first reduced the picture size to 20 by 20 pixels to standardize the image resolution and minimize computational complexity while preserving the essential color information (see Fig. 6 for an example). Next, we converted each non-background pixel’s RGB color to CIELAB coordinates. The CIELAB color space is designed to be perceptually uniform, meaning that a given numerical change corresponds to roughly the same perceived change in color, making it more aligned with human color perception (Fairchild, 2013). We then calculated similarities between all pairs of pixels across two objects using the CIEDE2000 color-difference formula. The CIEDE2000 formula, which operates within the CIELAB space, improves upon earlier color difference formulas by more accurately reflecting perceived differences between colors (Luo et al., 2001). Finally, we averaged the multiple pixel similarities across two objects to obtain the overall color similarity for each pair of comparisons.

We computed the Pearson correlation between the pairwise color similarity scores and the pairwise MDS distances to demonstrate the influence of color on similarity judgments. The results showed no significant correlation, *r*(433) = 0.04, *p* = 0.39, suggesting that colors did not bias participants’ similarity judgments.

#### Dimensionality

**Fig. 7:**
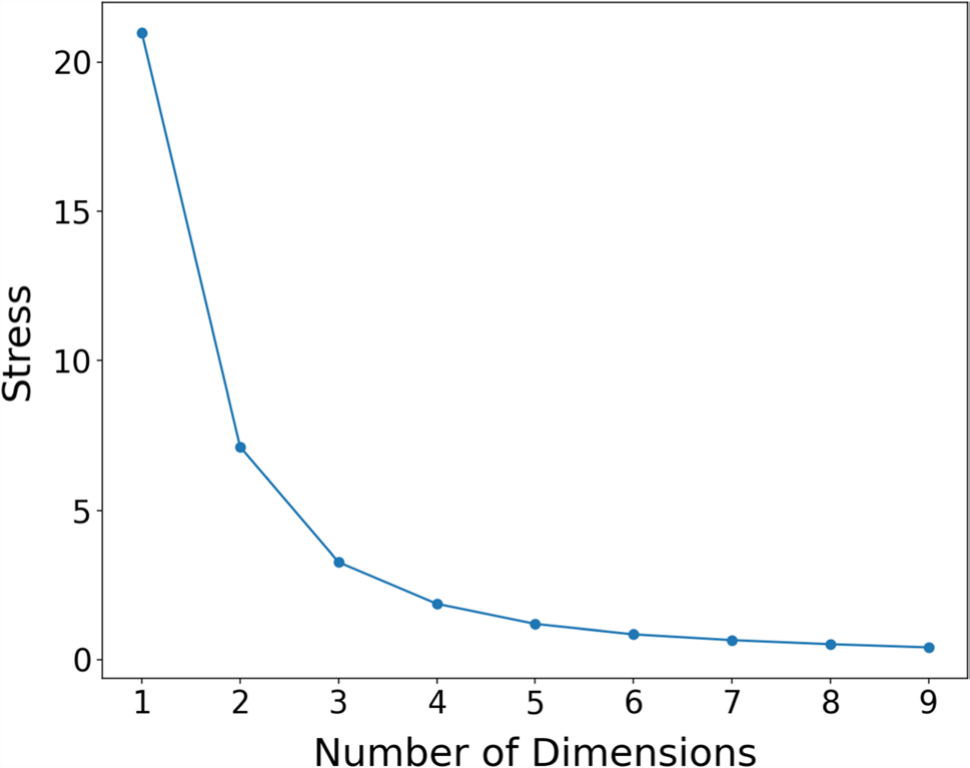
Scree plot. *Note*: This figure illustrates how stress values vary in relation to the dimensionality used in MDS. Each point represents the stress value corresponding to a specific number of dimensions in which the MDS data were scaled. Notably, a three-dimensional scaling appears to be optimal, as it balances the trade-off between a lower-dimensional representation and the preservation of the data structure, as captured by the stress metric in MDS

We constructed the scree plot shown in Fig. 7, where stress values appear to plateau at around three dimensions based on visual inspection. For an algorithmic determination of the optimal number of dimensions, we used “kneedle” as proposed by Satopaa et al. in 2011. The kneedle algorithm is primarily designed to work with curves exhibiting negative concavity, identifying the “knee” point where the increase of the *y*-value starts to plateau. However, in their original paper, Satopaa et al. (2011) also provided a solution for curves with positive concavity, which is relevant to the scree plots used in our analysis. This is achieved by inverting the knee graph, thereby enabling the identification of the “elbow,” which serves a similar purpose in positively concave curves as the “knee” does in negatively concave curves.

According to the results obtained from the kneedle algorithm, the “elbow” point on our scree plot is identified at three dimensions, consistent with the visual inspection. Notably, this is one dimension fewer than the result found in the NOUN database (Horst & Hout, 2016). This difference may be attributed to our methodology: when participants judge the similarity between objects, they are instructed not to consider color. This exclusion could account for the discrepancy in dimensions and suggest similar levels of dimensionality in the two databases. Nevertheless, judging solely based on shape still results in more than two dimensions, indicating that our objects are relatively complex.

#### Configuration

**Fig. 8:**
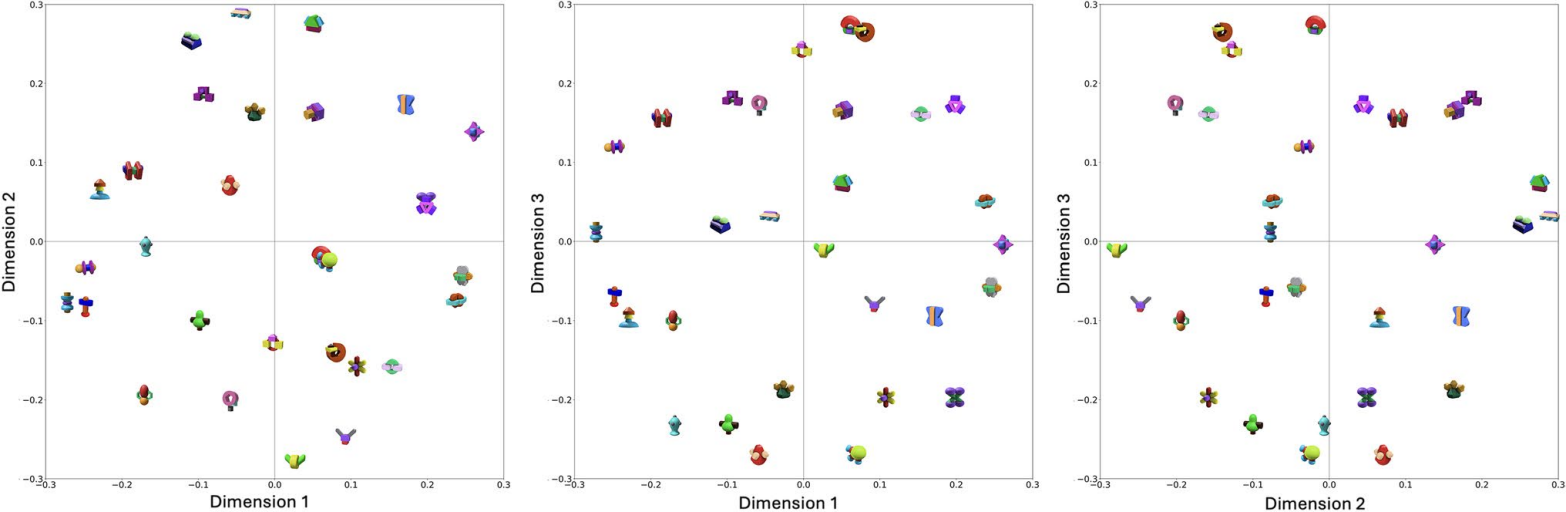
Visualization of MDS dimensions with object images superimposed on two dimensions. *Note.* From left to right: Dimension 1 vs. Dimension 2, Dimension 1 vs. Dimension 3, and Dimension 2 vs. Dimension 3, with the first dimension of each pair represented on the *x*-axis

We visualized the results obtained from the multiple-random-starts approach in a three-dimensional space, with object images superimposed for enhanced clarity. For a clear view, we first generated plots representing two dimensions at a time, as shown in Fig. 8.

**Fig. 9:**
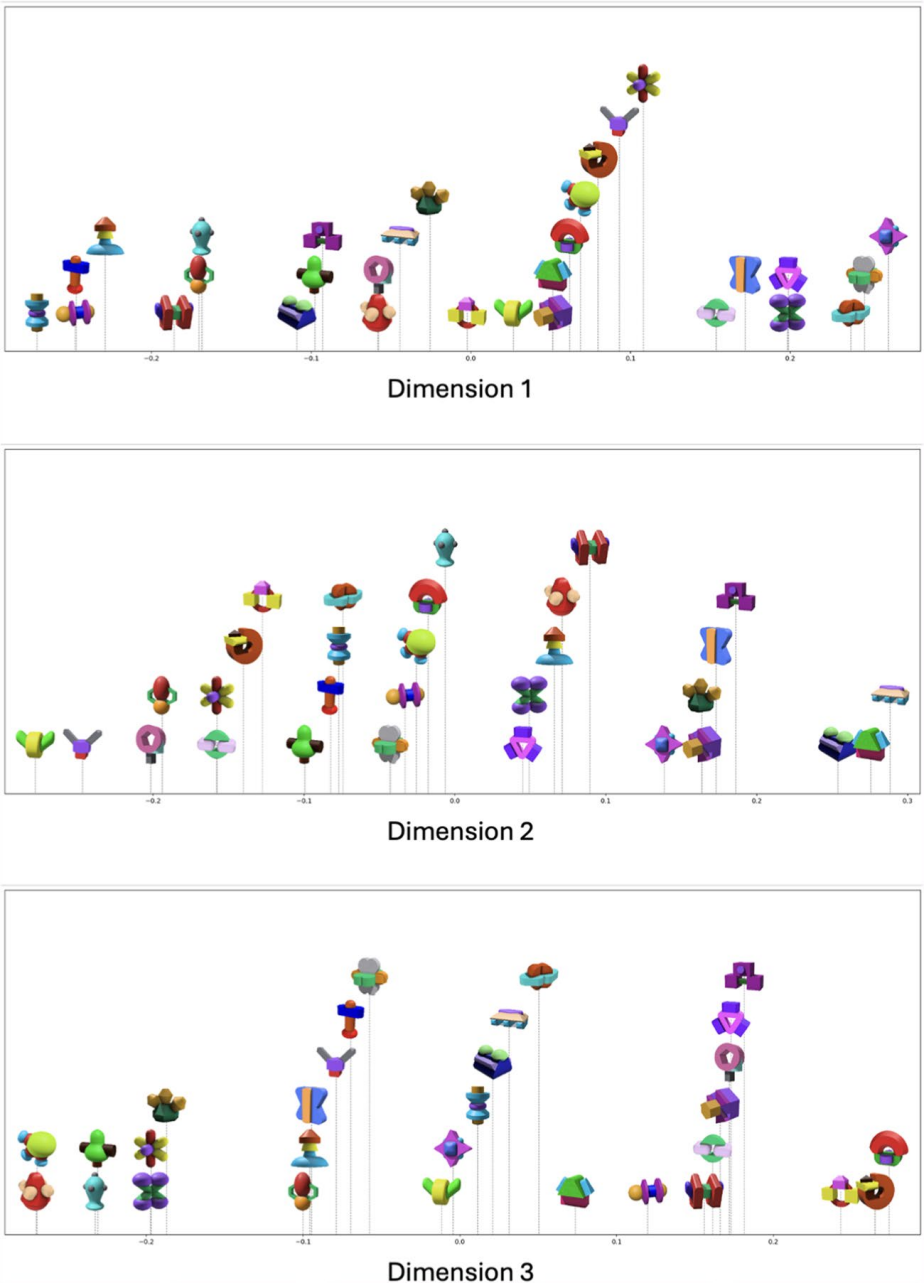
Visualization of MDS dimensions with object images superimposed on one dimension. *Note*. From top to bottom: Dimension 1, Dimension 2, and Dimension 3. The horizontal distance represents the distance between objects on a specific dimension. The vertical distances between objects are arbitrary and are plotted solely for enhanced visualization

Moreover, we generated plots in Fig. 8, each representing one dimension at a time, to see if it is possible to give interpretations to each dimension (i.e., axes). Although interpreting the axes from a brief summary remains challenging, we observe some clear patterns on each axis. For instance, in Dimension 1 (top graph in Fig. 9), the majority of objects positioned on the left exhibit an “elongated” shape, whereas those on the far right are more “rounded.”

#### Item pairs sorted by similarity

To provide researchers with a resource of item pairs varying in their degree of similarity (adopting a similar approach to that of Hout et al., 2014 and Horst & Hout, 2016), we rank-ordered the 435 item pairs based on their Euclidean distances in the three-dimensional MDS space (see Appendix C for the MDS distance matrix), from the smallest (most similar) to the largest (least similar). Subsequently, these item pairs were categorized into four quartiles. The first quartile represents the group of pairs that are generally the most similar, while the fourth quartile encompasses those that are the least similar. Table 1 summarizes the cutoffs for each quartile and sample pairs.

**Table 1:**
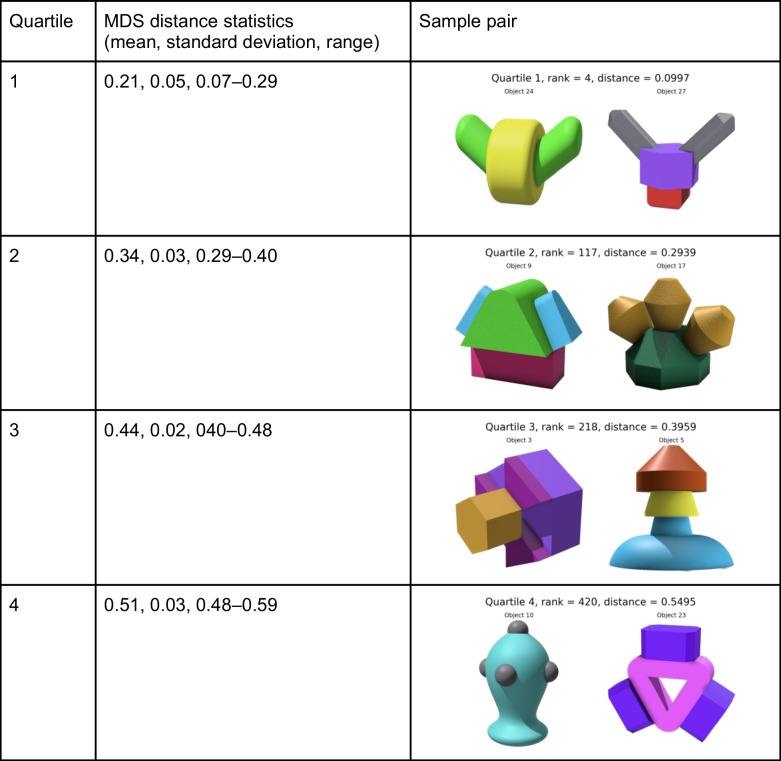
Quartile categorization of item pairs based on MDS distance

## Experiment 3

In Experiment 3, our goal was to define homogenous item subsets within the MDS space, termed “global categories,” through clustering analysis using MDS coordinates of MDS distances between object pairs found in Experiment 2. Our rationale for using measurements in the MDS space as the foundation for categorization aligns with well-established models in category learning, like the prototype and exemplar models of concepts. These models underscore the significance of similarity in relation to a category representation, highlighting how items within a category often share common features or attributes (refer to Murphy & Hoffman, 2012 for an extensive review).

In this experiment, we applied two distinct clustering methods: *k*-means clustering and hierarchical clustering. Each method addresses specific analytical objectives. *K*-means clustering ensures that each item is assigned to one distinct cluster, emphasizing exclusive categorization. In contrast, hierarchical clustering reveals nested (hierarchical) structures, allowing for the exploration of complex category relationships. The relevance of hierarchical structures is evident in everyday categories, such as the biological taxonomy of living organisms, underscoring the importance of understanding these structures. Research has demonstrated the impact of hierarchical structures on categorization behaviors (e.g., Medin et al., 1997; Voorspoels et al., 2011) and the mechanisms driving the formation of these hierarchies (e.g., Waxman, 1990). By incorporating novel hierarchical categories, we provide a controlled setting to explore how individuals develop and adjust hierarchical structures in categorization, thereby minimizing the influence of preconceived notions and biases often associated with familiar categories. This approach facilitates a clearer insight into the cognitive processes underpinning categorization.

Furthermore, we assessed the stability of the clustering results for each method and examined the concordance between them. This step ensures the robustness and reliability of our categorization, which is crucial for supporting further research into category learning and object complexity within the ALICE database.

### Method

#### *K*-means clustering

*K*-means clustering is an unsupervised machine learning approach that assigns data observations into nonhierarchical groups based on similarity/distance to others (Hartigan, 1975). It has been widely applied in multiple fields, including psychology (Steinley, 2006). This method partitions the dataset into *k* distinct, nonoverlapping (i.e., each observation belongs only to one cluster) clusters by maximizing the within-cluster homogeneity and between-cluster differences. The algorithm’s reliance on similarity/distance measures aligns with our methodological framework, leveraging the MDS coordinates derived from Experiment 2. This alignment ensures that our clustering approach is grounded on a consistent quantitative basis, facilitating the meaningful aggregation of objects into categories that reflect underlying similarities.

We conducted *k*-means clustering using Python’s scikit-learn package (Pedregosa et al., 2011). We submitted the raw coordinates of each object in the MDS space to the algorithm. These embeddings capture the essential spatial relationships between objects based on the original high-dimensional dissimilarities. To determine the optimal number of clusters (*k*), we constructed an elbow plot to visualize the mean squared Euclidean distance from each object to its assigned centroid in the MDS space (i.e., squared-error distortion) against the number of clusters. Furthermore, we validated the optimal number of clusters by comparing the “elbow” point (where increasing the value of *k* does not lead to a significant reduction in distortion) with the optimal number obtained from the kneedle algorithm (Satopaa et al., 2011).

#### Hierarchical clustering

To find the hierarchical structures among objects in the ALICE database, we conducted hierarchical clustering using Python’s SciPy package (Virtanen et al., 2020). This analysis produces a dendrogram, a tree-like diagram illustrating how objects are grouped into progressively larger clusters. These clusters demonstrate a nested structure, where any two clusters are either completely separate or one is entirely contained within the other (Hartigan, 2001).

We adopted the agglomerative approach to hierarchical clustering. This approach begins by treating each individual object as a distinct cluster, thus initially equating the number of clusters to the total count of objects. In subsequent steps, the algorithm iteratively merges clusters that are most similar to each other until all objects are eventually consolidated into a single, comprehensive cluster. The input argument for this analysis is the MDS distance metrics. We chose Ward’s method (Ward, 1963) as our clustering algorithm because it emphasizes minimizing the increase in total within-cluster variance at each step of the clustering process. This characteristic of the algorithm ensures that clusters are merged in a way that produces the smallest possible increase in internal variance, thereby fostering the formation of highly homogeneous clusters. Such clusters are desirable in our context, as they group objects that exhibit a high degree of similarity.

#### Stability test

To assess the robustness of our clustering methods and validate the quality of the resulting categories, we conducted a stability test for each algorithm. Stability tests are often conducted because unsupervised learning lacks true category labels against which to directly measure cluster performance (Liu et al., 2022), a situation that applies to our case, where true category labels are nonexistent for novel objects. To perform the stability tests, we employed bootstrap procedures to resample the data with replacement, which involves randomly selecting samples from our dataset with replacement. Specifically, we resampled the raw distance data obtained from each of the 96 participants in our study, thus creating 1000 bootstrap samples that each mirror the size of the original dataset (*N* = 96). Following the resampling, we conducted an MDS analysis on the complete dissimilarity matrix from each bootstrap sample. Subsequently, we applied each clustering method to the corresponding embeddings (MDS coordinates for *k*-means clustering and MDS distance matrix for hierarchical clustering), replicating the process used on the original dataset. Crucially, for both *k*-means and hierarchical clustering during the stability tests, the number of clusters was fixed at 7, based on the optimal clustering determined for *k*-means. This decision ensures consistency in evaluating the stability and comparability of the clustering outcomes.

The consistency of clustering outcomes was assessed using the Adjusted Rand Index (ARI), a widely used measure of agreement between two partitions (Santos & Embrechts, 2009), by comparing the bootstrapped clustering results against each other. A high ARI value between bootstrapped samples indicates that the algorithm consistently groups the same or similar items together across different subsets of the data, suggesting that the clustering structure is stable and not heavily influenced by the specific composition of the dataset. For each clustering method’s stability, we calculated an average ARI calculated from all pairwise comparisons. Additionally, to measure the agreement between the two clustering methods, we calculated the average ARI for the two methods across the entire dataset. The ARI was computed using Python’s scikit-learn package (Pedregosa et al., 2011).

### Results and discussion

#### *K*-means cluster analysis

**Fig. 10:**
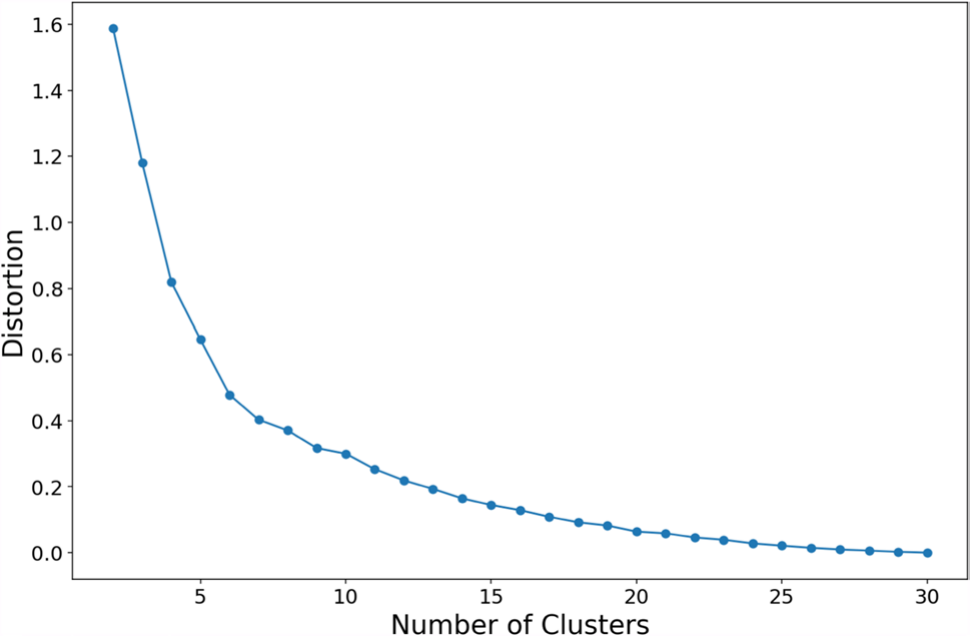
Elbow plot. *Note*: This figure illustrates distortion in relation to the number of clusters (*k*). Each point represents the distortion corresponding to a specific *k* value. Notably, we observed that an “elbow” appeared at *k* = 7

According to the elbow plot (Fig. 10), the decrease in distortion begins to slow down as the number of clusters increases beyond seven, indicating that adding more clusters does not significantly improve the model’s fit. Consistent with this visual inspection, the kneedle algorithm (Satopaa et al., 2011) identifies the “elbow” point at *k* = 7, confirming our observations.

**Fig. 11:**
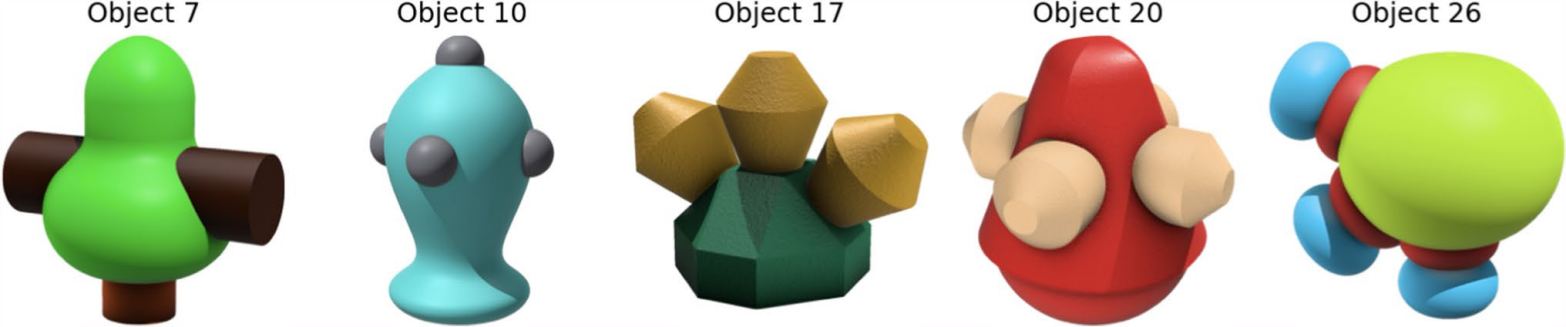
An example global category from *k*-means cluster analysis

The size of each resulting cluster ranged from three to six objects. We observe that objects within a particular category share specific features. For instance, as illustrated in Fig. 11, all objects in this particular category have some number of projecting parts. For category membership of all seven categories, see Appendix D.

#### Hierarchical cluster analysis

**Fig. 12:**
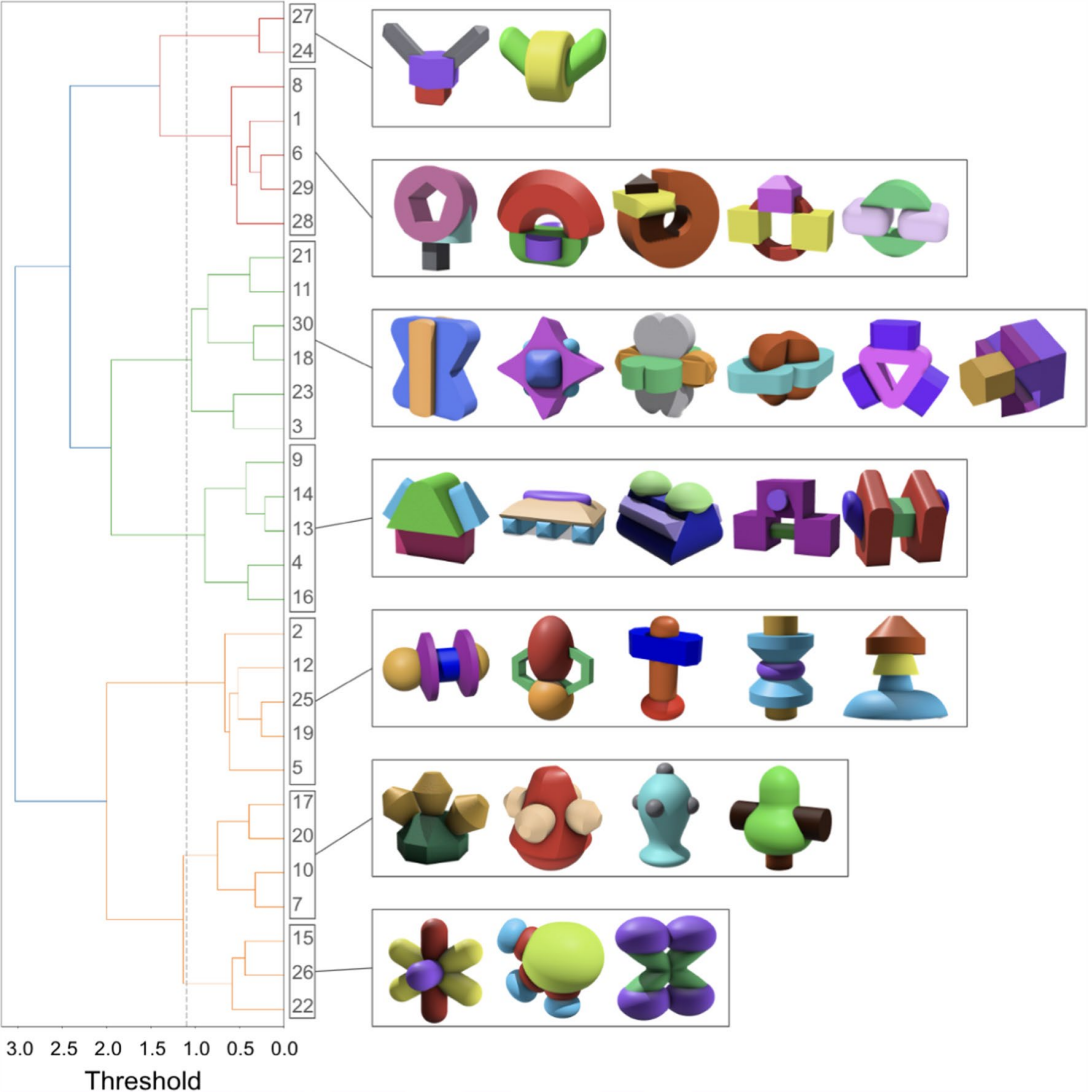
Dendrogram showing the hierarchical structure. *Note*: The threshold decreases from left to right along the *x*-axis. The numbers inside each vertical block are listed from top to bottom, corresponding to the objects in the clusters shown from left to right

A dendrogram (Fig. 12) was created by the hierarchical clustering. Categories are shown through vertical lines, and the *x* position of vertical lines shows the distance between the group members. That is, objects that are connected by lines at lower levels of the *x*-axis are more similar to each other than those connected at higher levels. For example, Objects 24 and 27 (Fig. 12, first-row cluster) are more similar than Objects 8, 1, 6, 29, and 18 (Fig. 12, second-row cluster).

We drew a vertical dashed line across the dendrogram that separated the objects into seven global categories, mirroring the number identified by the *k*-means clustering. This resulted in category sizes ranging from two to six, which is also comparable to the sizes obtained from *k*-means clustering. Notably, we observed a significant overlap in category membership. For instance, Objects 2, 19, 5, 25, and 12 were classified into the same global category by both methods.

#### Stability of clustering results and comparative analysis

We observed mean ARI values of 0.560 and 0.574 from the bootstrapped samples using *k*-means clustering and hierarchical clustering, respectively. These mean ARI values suggest that both methods exhibit moderate to good stability in their clustering outcomes when applied to bootstrapped samples.

Furthermore, a comparative analysis of the two clustering methods applied to the entire dataset reveals an average pairwise ARI of 0.641, suggesting moderate to good agreement between the two clustering methods. This ARI value attests to the reliability of our clustering techniques and validates the identified clustering structures, underscoring the effectiveness of our approach in delineating meaningful object categories.

In addition to clustering analysis on the MDS coordinates, we also explored the two clustering methods (*k-*means and hierarchical clustering) on the *t*-SNE coordinates derived from the three-dimensional *t*-SNE space. The resulting average ARI across the bootstrapped results from the two methods is very low at 0.108. This inconsistency suggests that *t*-SNE space might not sufficiently preserve the overall data structure. When data exhibits a clear global structure, various clustering methodologies can capture this structure with comparable effectiveness. Conversely, in scenarios where the data lacks a clear global structure, such as the *t*-SNE space (van der Maaten & Hinton, 2008), cluster identification becomes problematic irrespective of the clustering method employed. Consequently, different clustering algorithms may yield disparate results as they interpret the data distinctively based on their underlying principles and optimization parameters. Therefore, in our case, using MDS, which preserves global structure more effectively, resulted in more stable clustering outcomes across two clustering methods. This stability allows us to rely more confidently on the identified “global categories” from the MDS space. The complete analysis and results using the *t*-SNE space can be found in the supplementary analysis code at https://github.com/4lic3X/ALICE_database_analysis.

## General discussion

This research was motivated by the challenge researchers often encounter in preparing physical, novel objects for experimental purposes. In response, we introduced A Library for Innovative Category Exemplars (ALICE) database, an accessible and flexible tool designed to streamline stimuli preparation. A notable feature of the ALICE database is its adaptability—researchers can easily modify the objects’ size, shape, and texture to suit their experimental needs. The three experiments reported here validate different features of these objects: novelty, similarity, and global category.

The novelty of objects was confirmed by low agreement on naming/description (Experiment 1). The objects’ level of complexity is ensured by the number of dimensions based solely on shape (Experiment 2), making them comparable to real-life objects. Specifically, even without considering color and texture, we found that participants arranged the objects based on three primary dimensions. In addition, the similarity distances (Experiment 2) and the global categories (Experiment 3) can serve as useful guides for researchers when selecting stimuli based on their specific research questions.

A future direction is to gather novelty and similarity ratings from children. Due to differences in their life experiences, children may perceive novelty and similarity in significantly distinct ways compared with adults (Hammer & Diesendruck, 2005). Given that novel object learning tasks are often targeted at young children rather than adults, we acknowledge the inherent limitations of relying solely on ratings from adult participants in the current study. In the future, we plan to collect similarity measures from children using the printed objects.

In summary, the ALICE database provides an innovative solution to the challenges of producing novel physical objects. Its application can significantly save time and resources by offering sets of objects that are easy to produce and free researchers from the need for preliminary novelty and similarity analyses.

## Open Practices Statement

Data, materials, and analysis code are available at https://github.com/4lic3X/ALICE_database_analysis. None of the reported studies were preregistered.

## Data Availability

The datasets generated and/or analyzed during the current study are available in GitHub, accessible at https://github.com/4lic3X/ALICE_database_website (for data collection) and https://github.com/4lic3X/ALICE_database_analysis (for data and analysis code). Instructions for submitting STL files to the database are available at https://4lic3x.github.io/alice_stl/ . Additional supporting files are available from the corresponding author on reasonable request.

## Appendix A

Descriptive Statistics from Experiment 1

## Appendix B

Algorithm for Generating Blocks

The algorithm accepts two input parameters: N, the total count of distinct objects, and K, the number of objects to include in each trial. Its output comprises a list of constructed trials, delineating the number of required trials and the objects to display within each.

Initialization begins with three sets: P, encompassing all possible pairs of numbers from 1 to N (^n^C_2_ pairs); P*, an empty set for storing pairs covered by existing trails so far; and B, an empty set for holding the final output trails.

The algorithm proceeds with a loop, iterating randomly over pairs from P. The selected pair is denoted as p. Then, an empty set b is created to represent the current trial being generated. If p is not yet covered by a trial (i.e., if it is absent from P*), the numbers from p are added to b. Initially, the first number of the pair is added to b, and subsequently, the second number is included if b lacks sufficient objects (less than K). With the newly formed trial set b, all possible pairs among its numbers are generated and combined with P* to make these pairs as covered. The algorithm then proceeds to the next random pair from P until b contains enough objects. The trial b is then added to the result set B, and the process continues generating subsequent trial b until all pairs from P are processed.

## Appendix C

MDS Distance Matrix

Note. This figure presents the distance/dissimilarity matrix, constructed using normalized coordinates in the MDS space. Each cell in the matrix represents the dissimilarity value between a pair of items, with smaller values (indicated by darker shades) signifying greater similarity (i.e., shorter raw distance). The scale of dissimilarity is symmetric—hence, only the lower triangle of the matrix is displayed to avoid redundancy.

## Appendix D

Global Categories from K-means Cluster Analysis

## Code Availability

The code used for the analysis in this study is available on GitHub at the same links above.
